# A self-supervised framework for cross-modal search in histopathology archives using scale harmonization

**DOI:** 10.1038/s41598-024-60256-7

**Published:** 2024-04-27

**Authors:** Danial Maleki, Shahryar Rahnamayan, H. R. Tizhoosh

**Affiliations:** 1 Kimia Lab, University of Waterloo, Waterloo, ON Canada; 2 Nature-Inspired Computational Intelligence (NICI) Lab, Engineering Department, Brock University, St. Catharines, ON Canada; 3 Kimia Lab, Department of Artificial Intelligence and Informatics, Mayo Clinic, Rochester, MN USA

**Keywords:** Cancer imaging, Biomedical engineering, Computational science, Computer science

## Abstract

The exponential growth of data across various medical domains has generated a substantial demand for techniques to analyze multimodal big data. This demand is particularly pronounced in fields such as computational pathology due to the diverse nature of the tissue. Cross-modal retrieval aims to identify a common latent space where different modalities, such as image-text pairs, exhibit close alignment. The primary challenge, however, often lies in the representation of tissue features. While language models can be trained relatively easily, visual models frequently struggle due to the scarcity of labeled data. To address this issue, the innovative concept of harmonization has been introduced, extending the learning scheme distillation without supervision, known as DINO. The harmonization of scale refines the DINO paradigm through a novel patching approach, overcoming the complexities posed by gigapixel whole slide images in digital pathology. Experiments conducted on diverse datasets have demonstrated that the proposed approach significantly enhances cross-modal retrieval in tissue imaging. Moreover, it exhibits vast potential for other fields that rely on gigapixel imaging.

## Introduction

The rapid growth in data volume and variety, coupled with deep learning advancements, has led to real-world applications relying on integrating multi-modal data^1,2^. These applications demand comprehensive data from various sources, but current models typically handle only one data type, limiting their versatility. Meeting the rising need for multi-modal models is crucial in academia and industry. These models process diverse data, enhancing decision-making and efficiency across sectors.

Single-modal search, e.g., image search, has been approached via search engines like Yottixel^3,4^. Multimodal learning integrates data from various types like text, images, and audio^5^. It aims to comprehend and link diverse data, providing comprehensive insights by merging different modes. Cross-modal retrieval, a specialized form of multimodal learning, fetches entities from one modality using a query from another, presenting unique challenges in bridging semantic gaps and managing inconsistencies across modalities. Both multimodal learning and cross-modal retrieval contribute to understanding and integrating diverse data for decision-making. Cross-modal retrieval, gaining popularity, uncovers connections between data from different sources, crucial for applications like medical diagnosis, recommendations, and multimedia search engines.

Designing cross-modal networks is challenging as they must learn inter-modal correspondences and individual modality representations^6,7^. Images and text are common modalities, often conveying complementary information. Cross-modal retrieval models leveraging this data offer nuanced contexts, enhancing information retrieval. These models integrate diverse data sources, enabling generalizable problem representations, improving decision-making in various tasks. Extracting meaningful embeddings from sparsely labeled data, especially in complex domains like medicine, is crucial. Cross-modal networks that learn from diverse data sources without heavy reliance on labeled data have transformative potential. In healthcare, these models could integrate diverse patient data, aiding comprehensive understanding and informed decision-making, revolutionizing patient care.

Cross-modality retrieval tasks, involving matching descriptions to images or vice versa, are a recent area of study. Early efforts like MDNet^8^ mapped images to diagnostic reports but faced limitations with small private datasets. Gamper et al.^9^ developed the ARCH dataset, utilizing medical articles and textbooks, amassing 7000+ image-caption pairs. Other studies explored similar tasks in pathology using innovative techniques, leveraging large-scale internet data. However, skepticism arises due to potential data quality issues. Despite recent advancements, models trained on high-quality datasets can show a better performance in some specific tasks^10^ .

Cross-modal data retrieval and fusion enhance decision-making in diverse fields. Robust models that handle various data types are crucial as data volume rises. Advancements in cross-modal retrieval are essential to fully leverage multi-modal data, ensuring improved outcomes in real-world scenarios.

The common solution to understand the relationship between image and text is to map the visual semantic embeddings^11,12^ of an image and the corresponding words, phrases, sentences into a *common latent embedding space*^11,13–18^. In these methods, the goal is generally to find a common space in which the corresponding representations of image-text pairs are as close as possible, hence making the recognition of their relationship easier.

To gain a better understanding of the similarity between the two modalities, recent studies investigate the use of the attention mechanism. The term “*attention*” refers to a process that simulates cognitive attention to highlight the most relevant features of the input data while fading the rest. Attention-based topologies assume that the model, generally an artificial neural network, should dedicate additional computational resources to that minor but significant portion of the data. Which part of the data is more significant than others is determined by the context and can be learned by gradient descent on training data^19^. The majority of studies in this category have employed cross-attention mechanisms, which allow the model to selectively attend to the parts of an instance that are relevant to the context from the other modal^7,20,21^. Some methods attempt to refine their representation regarding the information of the other modality^6^.

Nonetheless, these methods may not be able to find the optimal representation due to the semantic gap between the representation of images and texts. Moreover, ambiguity in texts is a common challenge posing a learning obstacle to the model if it uses an injective embedding^21^. Aside from that, humans use a hierarchical structure to organize and store diverse semantic concepts. However, the majority of the currently available approaches group semantics together in a consistent manner^7,21^. An approach for observing semantic concepts in a hierarchical framework that captures low-level features first and, subsequently, higher-level features could mimic human intuition more realistically. Another challenge, as mentioned above, is the difficulty and expense associated with obtaining *labeled data*. This makes extracting meaningful embeddings a considerable hurdle. To tackle this issue, some methods have explored the self-supervised learning paradigm^22^. The majority of these approaches define a *pretext task* based on unlabeled inputs to generate informative and comprehensible representations^23,24^. However, designing these pretext tasks requires a different approach due to the distinct nature of histopathology data and its formatting as Whole Slide Image (WSI).

This study addresses the mentioned problems by introducing an iterative regime that not only captures related information from the other modality but also extracts the most significant attributes by considering their context. This approach enables the model to simultaneously capture information relevant to both modalities, leading to the extraction of richer latent embeddings for each instance. Moreover, training the model iteratively allows it to gradually capture higher-level features based on those acquired in previous steps. Refining the extracted features through the intersection between modalities further enhances the model’s representations.

To overcome the obstacle of labelled data scarcity, particularly for gigapixel images, this study proposes a self-supervised approach utilizing a novel patching scheme. This scheme aims to adapt to the intrinsic features of WSI and assist the model in generating more robust embeddings for the visual component. The entire model is trained using an end-to-end regime, which improves the accuracy and efficiency of the model^25,26^.

Building on the foundation of addressing these critical issues, this study introduces a concept that merges SSL techniques, specifically leveraging the DINO approach^27^, with a novel patch sampling strategy that is uniquely suited for WSIs. This strategy is further integrated with cross-modality retrieval network and the unique design of it enables the capability of an end-to-end training paradigm.

Extensive experiments have been carried out on public datasets including PatchGastricADC22^28^ and LC25000^29^, as well as a private dataset from GRH (Grand River Hospital, Kitchener, ON) to evaluate the effectiveness of the proposed method. these datasets consist of histopathology images along with their corresponding captions/reports/primary diagnoses, which are extracted from histopathology textbooks, the PubMed repository^30^, originate from clinical practice in GRH case.

## Results

A comprehensive evaluation of the proposed method in comparison to alternative approaches was undertaken by applying two strategies tailored to the specific nature of the datasets. The datasets in question, namely GRH and LC25000, encompass images that have been classified according to primary diagnosis and can serve as a definitive classification criterion. This can help us to compare the proposed approach with other models like KimiaNet^31^. As a result, in the conducted experiments on these datasets, the primary diagnosis was utilized as a descriptor for the proposed method, and to facilitate a fair comparison with other techniques without compromising the task’s generality, the primary diagnosis was mapped into labels compatible with other classification approaches.

Moreover, the proposed method was demonstrated to function as a *bi-directional retriever* for both GRH and LC25000 datasets by feeding the primary diagnosis as a description into the model. This allowed an evaluation of its performance in different settings, offering a more robust and comprehensive assessment of its efficiency and applicability.

In relation to the PatchGastricADC22 dataset, which is characterized by paired image-description entries at the WSI level, the provided descriptions were employed as text inputs. This was informed by the unique structure and specific attributes of this dataset, which is amenable to a deeper level of analysis courtesy of the substantial data encapsulated within the descriptions. Consequently, other existing cross-modal retrieval methodologies were used as points of reference for comparison and evaluation. This approach ensured that the evaluation benchmark remained constant, facilitating a rigorous comparison rooted in the specific characteristics of the dataset.

The versatility and adaptability of the proposed method to different dataset configurations were highlighted, further validating its utility in various machine-learning scenarios.

In the subsequent sections, a thorough delineation of the procedural specifics associated with the methodology’s implementation is provided, in addition to an in-depth presentation of the experimental results derived from each of the datasets. The aim of these discussions is to illuminate the complexities of the proposed approach, underscore its practical utility, and confirm its resilience when applied to diverse datasets and configurations.

### GRH benchmark dataset

To conduct experiments on the GRH dataset which contains 173 WSIs , obtained from a wide-ranging patient population diagnosed with an array of breast cancer subtypes at the GRH. These primary diagnoses are classified into 22 distinct categories, marking the broad spectrum of breast cancer sub types. Rigorous pre-processing steps were employed, where patches of size 448 by 448 pixels in 20X magnification were extracted from WSIs based on Figure 9. The student model was trained using six stringent data augmentation techniques ($\tau _H$), whereas the teacher model had a relatively relaxed regiment with two less intensive augmentations ($\tau _M)$ from the same patch as will be described in the patching Section. Regardless of the specific augmentation technique implemented, the final processed image was adjusted to fit a uniform size of 224 by 224 pixels. Padding was added when necessary to ensure a consistent dimension across all images. In the cross-modal network the center crop of each patch has been selected as the input to the model. A similar augmentation set as $\tau _M$ is applied on this input for uniformity. The suite of augmentations applied to this dataset encompasses a range of transformations. These include Random Crop, Random Vertical and Horizontal Flip randomly at $p=0.5$, as well as Color Jitter, which further encompasses changes in Brightness, Contrast, Saturation, and Hue. The intensity of these augmentations is strategically regulated, being categorized under either Hyper or Mild augmentation sets based on the severity of the transformation required. For the image representation extraction and SSL component, the student and teacher models utilized a Vision Transformer (ViT) architecture with 12 Transformer encoding blocks and 768 for the hidden states. Weight initialization for both student and teacher networks was facilitated through a pre-trained ViT model using the dataset featured in the “KimiaNet” paper^31^. The last six blocks of the student and teacher models are set to be trainable and frozen the rest.

The text encoder component utilizes a robust network built on BERT, featuring 12 Transformer layers. Weight initialization for both the student and teacher text encoders leverages BioBert^32^ to ensure optimal performance, with fine-tuning restricted to the last two blocks. An empirical approach led to the determination of optimal values for $\beta$ and $\gamma$ in Eq. (8), where the most desirable performance was observed at $\beta = 0.3$ and $\gamma = 1$. This configuration subtly de-emphasized the SSL component within the loss function. Given that the primary objective of the function is to identify and match paired images and texts, this adjustment allowed the model to focus more on its primary task while still benefiting from the auxiliary guidance of the SSL component.

To mitigate any potential bias and maintain the fairness of the comparison, six experimental iterations were carried out, each with a different test dataset, and assigned the rest of the dataset as train and validation sets. To further validate these comparisons, it was ensured that each test set included all 22 primary diagnoses. Model training was accomplished using the AdamW optimizer^33^, with an initial learning rate of $1e-6$. The learning rate was adapted to decay by a factor of 0.5 when the evaluation metric (R@sum) ceased to improve. The configuration for the rest of the model remains consistent with the optimal parameters identified in the LILE experiments^34^. This includes the number of iteration steps (denoted as *K*) and the weight factor (represented by $\alpha$) within the loss function. The selection of these parameters is driven by the previous findings, thus ensuring that the model is effectively tuned for peak performance. This configuration continuity allows for a more robust evaluation and comparison across datasets. A comprehensive account of the findings from this setup can be found in Table 1.

**Table 1:** comparison among the proposed method and other state-of-the-art approaches, examining their performance in both patch-based and WSI-based retrieval tasks on the GRH dataset.

Method	Text retrieval				Image retrieval				
	R@1	R@3	R@5	R@10	R@1	R@3	R@5	R@10	R@sum
Patch based									
KimiaNet^31^	$24.1 \pm 3.6$	$43.3 \pm 2.3$	$51.2 \pm 2.2$	$72.3 \pm 1.9$	N/A	N/A	N/A	N/A	N/A
BioMedCLIP^35^	$12.6 \pm 1.4$	$31.1 \pm 3.7$	$42.7 \pm 3.9$	$66.7 \pm 5.3$	$18.4 \pm 1.7$	$34.0 \pm 3.2$	$41.9 \pm 3.6$	$60.2 \pm 4.2$	$307.6 \pm 10.3$
LILE^34^	$29.3 \pm 4.3$	$50.0 \pm 3.5$	$63.6 \pm 2.0$	$80.2 \pm 1.9$	$39.2 \pm 4.7$	$53.6 \pm 4.2$	$57.5 \pm 4.6$	$61.5 \pm 3.1$	$434.9 \pm 15.1$
LILE + DINO	$31.6 \pm 4.2$	$53.8 \pm 3.1$	$65.4 \pm 2.5$	$81.9 \pm 1.0$	$40.1 \pm 6.7$	$57.6 \pm 5.6$	$63.9 \pm 4.8$	$64.8 \pm 3.1$	$459.1 \pm 23.7$
LILE + H − DINO	$\mathbf {31.9} \pm \mathbf {3.7}$	$\mathbf {54.2} \pm \mathbf {3.1}$	$\mathbf {65.8} \pm \mathbf {2.4}$	$\mathbf {82.3} \pm \mathbf {1.0}$	$\mathbf {43.2} \pm \mathbf {6.8}$	$\mathbf {59.8} \pm \mathbf {4.1}$	$\mathbf {64.4} \pm \mathbf {4.8}$	$\mathbf {65.9} \pm \mathbf {3.5}$	$\mathbf {467.5} \pm \mathbf {18.8}$
WSI based									
KimiaNet^31^	$42.3 \pm 5.3$	$66.3 \pm 3.2$	$70.4 \pm 3.0$	$78.1 \pm 2.1$	N/A	N/A	N/A	N/A	N/A
BioMedCLIP^35^	$32.4 \pm 2.1$	$48.4 \pm 3.2$	$68.3 \pm 3.4$	$74.1 \pm 3.0$	N/A	N/A	N/A	N/A	N/A
LILE^34^	$50.0 \pm 6.1$	$70.1 \pm 4.2$	$76.4 \pm 2.4$	$81.8 \pm 2.9$	N/A	N/A	N/A	N/A	N/A
LILE + DINO	$53.7 \pm 7.3$	$75.7 \pm 4.3$	$83.2 \pm 3.1$	$90.9 \pm 2.6$	N/A	N/A	N/A	N/A	N/A
LILE + H − DINO	$\mathbf {54.5 \pm 6.4}$	$\mathbf {77.3 \pm 4.5}$	$\mathbf {84.1 \pm 2.3}$	$\mathbf {92.5 \pm 3.3}$	N/A	N/A	N/A	N/A	N/A

Best results highlighted in bold.

The experiments on the GRH dataset were conducted in two different schemes. As shown in Table 1, the proposed method is compared with other methods in **patch-based** and **WSI-based** configurations. For the patch-based strategy, each patch needs to predict the correct primary diagnosis related to itself. In an alternate setup, the problem was approached through a WSI retrieval lens, replacing the individual patch-based retrieval. The strategy being used places considerable emphasis on **majority voting** for the prediction of the WSI primary diagnosis. This approach, which is largely derived from strategies employed in patch-based experiments, is centred around the accumulation of predictions made for individual patches. The process unfolds as follows: for each patch, a determination is initially made regarding whether it has accurately retrieved its correct primary diagnosis, with various thresholds applied in the recall metric. If the true primary diagnosis is found among the top “K” retrieved diagnoses, the patch is identified as correctly retrieving the actual text. If not, the patch is assigned the label corresponding to the first retrieved primary diagnosis. Subsequently, a label is assigned to a WSI, reflecting the most common label among its constituent patches. This strategy provided a more holistic interpretation of the WSI, as it accounted for the collective intelligence of all patches in a WSI, rather than treating each patch in isolation. This perspective shift aimed at enhancing the overall retrieval performance by considering the consensus of predictions within a WSI, thus adding another dimension of robustness to the proposed method.

In the analysis of KimiaNet, a pre-trained model that had been subjected to comprehensive training using the vast TCGA dataset^36^ was employed. This model underwent further fine-tuning using the training data, with the parameters of the last fully connected layer set to be trainable. For the experiments conducted on BioMedCLIP^35^, their pre-trained model without any additional fine-tuning is utilized. Their model was trained on 15 million pairs of images and text extracted from articles published on PubMed.

The proposed method, incorporating structure into its backbone and employing the H-DINO strategy for self-supervision, demonstrated superior performance in both patch-based and WSI-based tasks compared to other approaches. A comparative analysis, pitching the proposed LILE + DINO method against the without any self-supervision, demonstrated the potency of SSL in boosting model performance. The inclusion of SSL significantly elevated the R@sum metric, which measures the model’s overall effectiveness, by a notable 32.6 margin. Furthermore, a comparison drawn between the LILE + H − DINO and the LILE + DINO methods underscored the value of implementing a patching method specifically devised for WSIs. This tailored approach to patching further enhanced the R@sum performance metric by an additional 8.4, highlighting its crucial role in optimizing the model’s overall performance. This specialized approach notably improved the model’s performance by providing a more robust embedding representation for the patches. An independent samples t-test was conducted to assess the differences in performance between the proposed LILE + H − DINO method and LILE + DINO. The analysis yielded a t-statistic of 3.6 and a *p*-value of 0.00033, indicating that a statistically significant improvement in the R@sum metric was observed with LILE + H − DINO over LILE + DINO. This result compellingly demonstrates the superior efficacy of LILE + H − DINO. Furthermore, the study presented in LILE paper^34^ demonstrated that the LILE model surpassed the performance of the CLIP model^37^. Consequently, the CLIP model’s results were omitted from reporting in subsequent experiments.

When considering text-to-WSI retrieval, the nature of the training and testing data, which are primarily patches, made it unfeasible to retrieve a WSI based on its primary diagnosis. This is due to the inherent fact that all patches extracted from the same WSI share an identical primary diagnosis, leading to a uniform result. Consequently, no results have been reported in this direction, and this aspect is noted as “N/A” (Not Applicable). Further, it is worth mentioning that the KimiaNet and other models exclusively trained for images lack the capability to retrieve images when given text, leading to an absence of reported results for these models. However, within the scope of patch-based retrieval, the models that are built upon display the compelling feature of bidirectional functionality. This characteristic highlights the distinct superiority of cross-modal retrieval models over traditional classification networks, such as KimiaNet, as they possess the capacity to operate fluidly in both retrieval directions.

It should be noted that WSIs are predominantly utilized for diagnostic purposes by medical practitioners, including oncologists and pathologists. Consequently, greater emphasis is placed on the performance of the model in the WSI-based approach as compared to patch-based results. As seen in the table, the results for the WSI-based approach considerably outperformed those for the patch-based method. This phenomenon could be attributed to the power of majority voting. A WSI was assigned to a label if the majority of its patches voted for that specific label, implying that even if some patches were incorrectly predicted, the overall true label could still be accurately identified.

**Figure 1:**
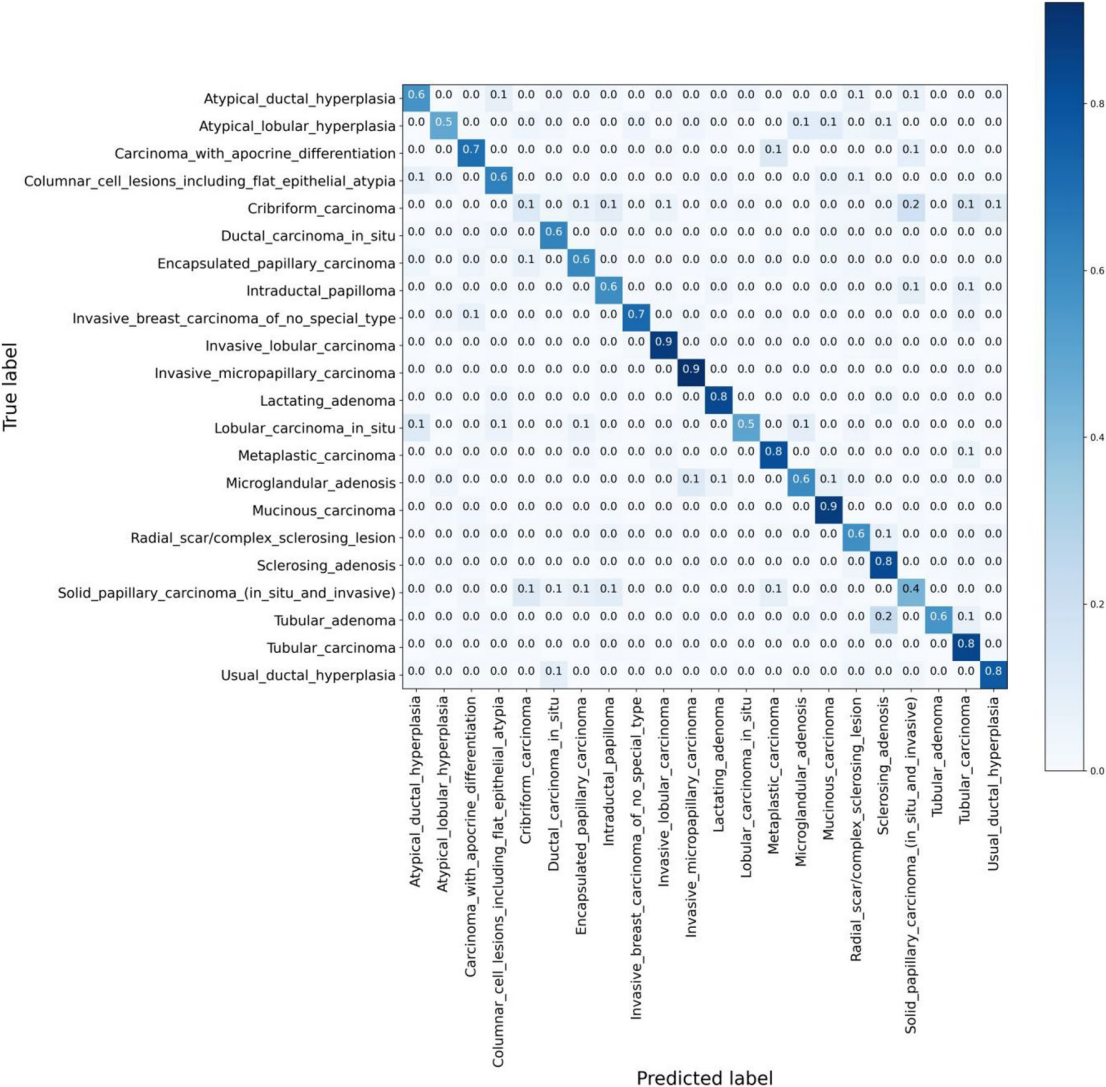
This confusion matrix depicts the retrieval performance of the proposed method for **patch-level** classification across 22 distinct primary diagnoses, specifically at an **R@5** recall level. It provides a comprehensive visual representation of how accurately the model classifies each diagnosis and demonstrates how the results are distributed across true and predicted classifications, hence offering a deeper understanding of the model’s performance.

**Figure 2:**
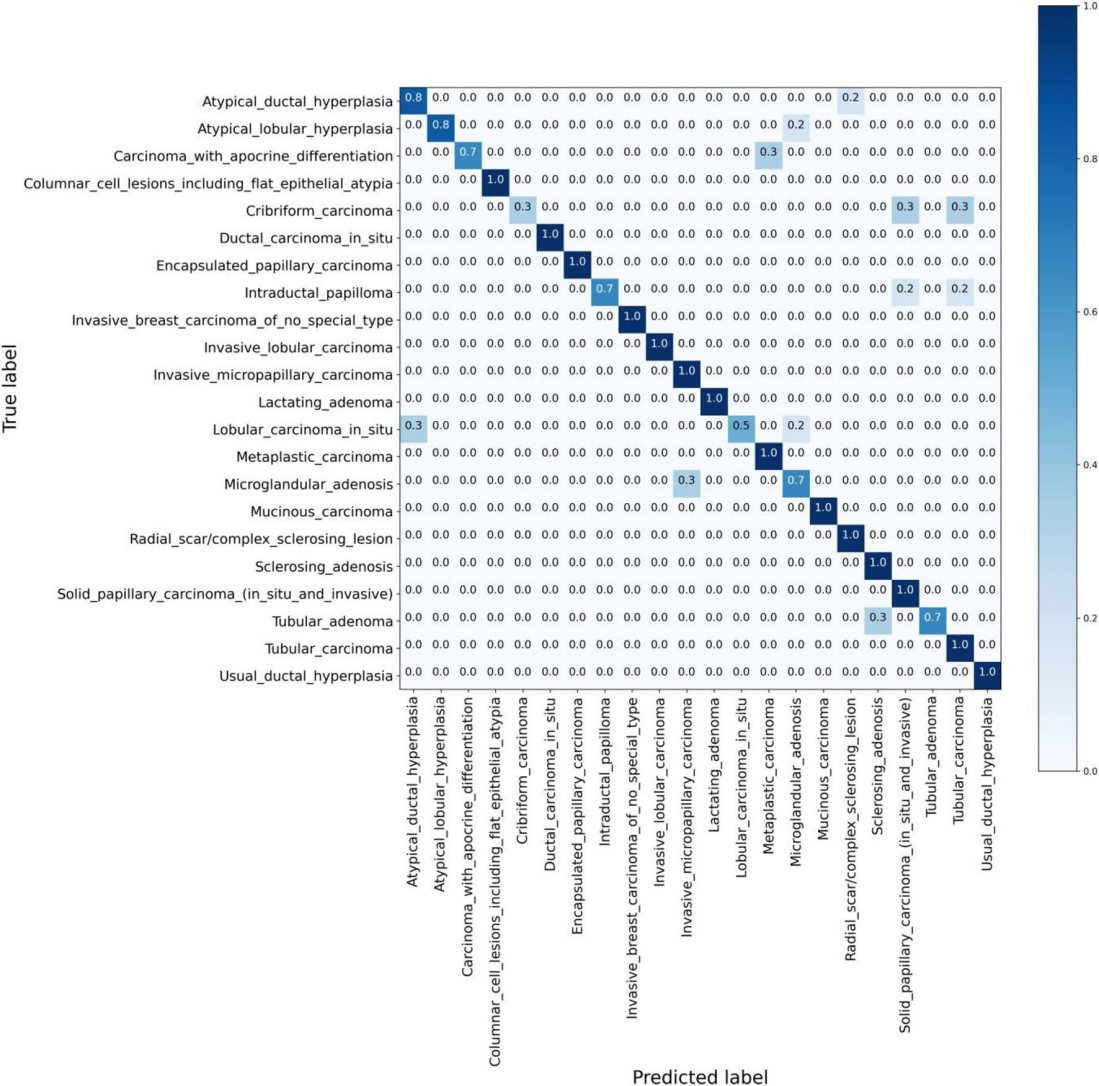
This confusion matrix provides a visualization of the retrieval performance at the **R@5** level of the proposed method for the **WSI-level** classification across 22 distinct primary diagnoses. It serves as a detailed display of the model’s accuracy in diagnosis classification. By presenting the distribution of results across true and predicted classes, it enables a profound understanding of the overall efficacy and precision of the model.

To further corroborate the efficacy of the proposed method, confusion matrices for R@1, R@3, and R@5 in patch-based strategy are presented in Fig. 1. Moreover, the comprehensive performance of the model in the context of WSI retrieval at various recall thresholds – R@1, R@3, and R@5 – is visualized in confusion matrices, as depicted in Fig. 2. To generate the confusion matrices that are displayed, the same methodology described in relation to Table 1 is employed. This approach allows for a consistent and comparative representation of the data across different visualizations.

These figures enable an in-depth understanding of the model’s capability to assign diagnoses at different levels of recall accurately. From the confusion matrices can be understood that the proposed approach can distinguish more of its classes, and by going from R@1 to R@3 and R@5, i.e., looking at more retrievals, one can achieve more reliable predictions. Classifying 22 primary diagnoses of breast cancer is extremely challenging as they are all related to one anatomical organ, and they exhibit many similarities in their texture. As a result, R@3 and R@5 can provide more accurate results and be practical for pathologists and doctors to be relied on. Furthermore, it is noteworthy that each primary diagnosis can occasionally be misconstrued as a different primary diagnosis. This phenomenon could potentially serve as an additional source of information for pathologists. By allowing for the possibility of alternative diagnoses, insights that might be obscured if only the correct primary diagnosis were provided can instead be unveiled, thus supporting a more comprehensive understanding of the pathology in question.

**Figure 3:**
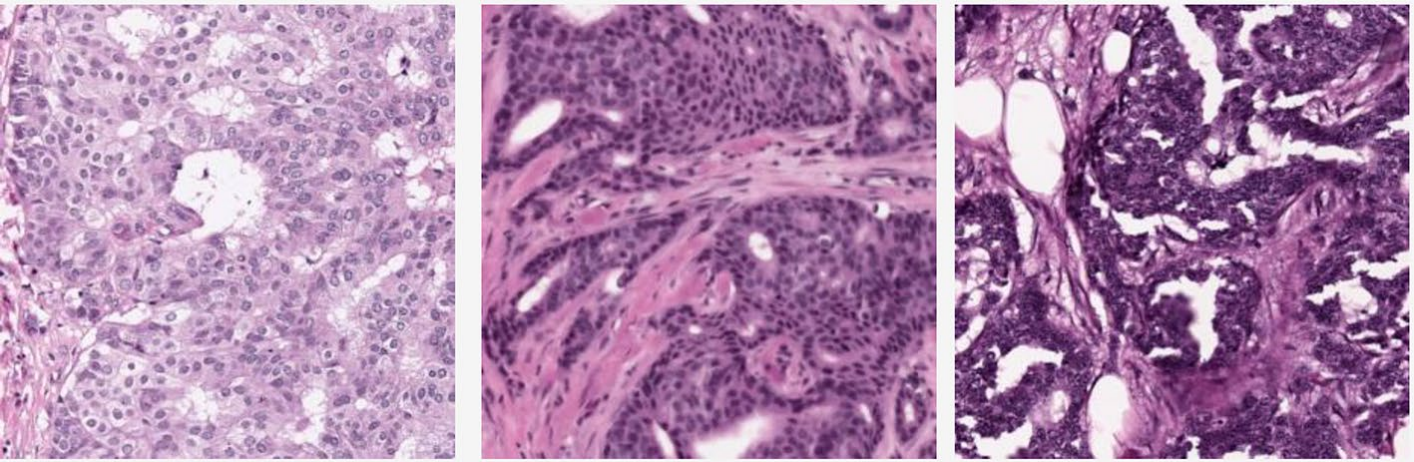
Three randomly selected patches from three distinct Cribriform Carcinoma WSIs present in the GRH dataset. The noticeable differences in structure and the significant variations among these images illuminate the challenge of accurate classification, ultimately contributing to lower accuracy rates.

More examination of the confusion matrices reveals that two primary diagnoses, namely, *Caribiform Carcinoma* and *Lobular Carcinoma* in Situ, pose significant challenges. A contributing factor to the misclassification of Caribiform Carcinoma pertains to the limited representation of this diagnosis in the GRH dataset. With only three WSIs corresponding to this diagnosis, the dataset configuration for each training run is such that one WSI is set aside for testing, and another for validation, leaving just a single WSI for training. As depicted in Fig. 3, which showcases three random patches extracted from these three WSIs, the structural and feature variation across each WSI is quite significant. Consequently, training the model using a single WSI fails to provide a comprehensive representation of the primary diagnosis, thereby making it challenging to correctly identify this diagnosis in other WSIs.

If more data corresponding to this specific primary diagnosis were to be included in the training set, it would be reasonable to anticipate improved diagnostic accuracy. By enhancing the diversity and volume of the training data, the model would be equipped with a more holistic understanding of the diagnosis, thus improving its ability to identify and classify new instances accurately.

**Figure 4:**
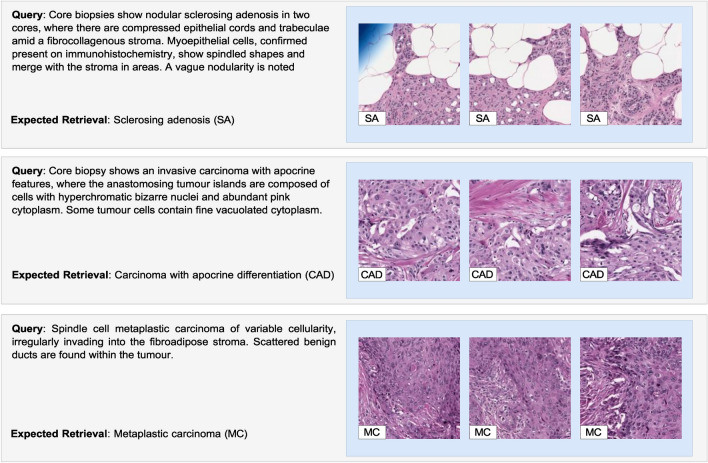
Visualizing image retrieval using real-world medical descriptions extracted from the WHO dataset, which wasn’t included in the original training set. Under each description, the associated primary diagnosis is displayed. To the right, the top three images retrieved by the model are presented alongside their corresponding primary diagnoses. This illustration showcases the model’s ability to retrieve and match images based on textual medical descriptions accurately.

In another experiment, the robustness and adaptability of the proposed model were demonstrated through an analysis of the generated embeddings and the real-world application of these findings. The dataset used for this experiment was sourced from the World Health Organization (WHO), which was not part of the original training set but provided real medical reports all related to the same primary diagnosis.

The top three results from the text-to-image (t2i) retrieval process are displayed in Fig. 4. The capability of the model to retrieve accurate and relevant images given a description of a specific primary diagnosis is clearly illustrated by these results. These examples demonstrate how the model can identify and understand similar semantic structures between images and their corresponding descriptions. Not only does this enhance the overall precision of the model, but it also demonstrates its ability to adapt to and comprehend new, intricate concepts.

**Table 2:** Comparative analysis of **WSI-based** performance on the GRH dataset using Recall@K@V metric with a “V” threshold of five—illustrating the superior performance and adaptability of the proposed LILE + H − DINO method across diverse recall thresholds.

	R@1	R@3	R@5	R@10
KimiaNet^31^	$43.0 \pm 5.0$	$63.6 \pm 2.3$	$69.1 \pm 2.5$	$78.5 \pm 2.3$
LILE^34^	$50.8 \pm 6.2$	$70.0 \pm 5.6$	$76.0 \pm 1.8$	$81.5 \pm 2.4$
LILE + DINO	$56.6 \pm 5.5$	$72.5 \pm 5.4$	$80.9 \pm 3.2$	$89.9 \pm 3.5$
LILE + H − DINO	$\mathbf {57.2} \pm \mathbf {5.2}$	$\mathbf {74.1} \pm 4.7$	$\mathbf {81.8} \pm 4.0$	$\mathbf {91.9} \pm 4.0$

Best results highlighted in bold.

In additional sets of experiments conducted on the GRH dataset, a focus was placed on evaluating the model based on metrics more aligned with the diagnostic considerations of pathologists in order to ensure real-world clinical applications were closely reflected. One of the key metrics, recall@K (R@k), which was reported previously, was utilized for this evaluation. The successful retrieval was defined by the premise that the correct item was found within the top K retrieved items. This measure of success was then used to cast a ‘vote’ among the patches of a WSI.

Taking the approach a step further, another strategy was adopted. This method involved the top “V” retrieved primary diagnoses for each image patch being gathered. Here, “V” functions as a voting threshold, representing the number of retrieved items deemed informative enough to contribute to the majority vote in the ultimate step. Subsequently, these items were multiplied by their corresponding normalized similarity scores. The primary diagnoses retrieved for each WSI were then organized based on the frequency of their occurrence among the top “V” diagnoses, which were weighted by their scores across all patches within that specific WSI. Following this, the recall@K evaluation was applied to ascertain if the accurate primary diagnosis was among the top “K” retrieved items. This layered evaluation technique, termed **Recall@K@V**, provided a multidimensional and more nuanced analysis of retrieval success and presented a more comprehensive picture of retrieval performance. Such a metric can be of assistance to pathologists when ordering immunohistochemistry; Recall@K@V is a good quantification to select the right biomarkers.

**Figure 5:**
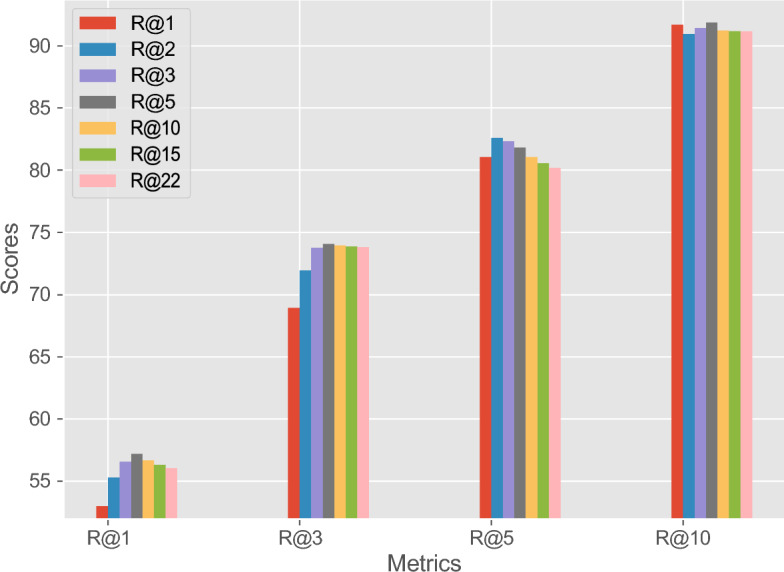
This figure illustrates the Recall@K@V values for various combinations of V and K. In this process, the most relevant primary diagnoses are first sorted for each patch based on a given V value. Subsequently, the Recall@K is computed for each WSI using the frequency of the most common primary diagnosis identified for that WSI. The results of this process are showcased in the figure for multiple voting thresholds (V), which include 1, 2, 3, 5, 10, 15, and 22. The selection of these thresholds is significant as the number of primary diagnoses is capped at 22. Additionally, the figure also displays these results across varying levels of Recall (K), specifically 1, 3, 5, and 10. This visualization provides a comprehensive overview of how alterations in the voting threshold and recall level influence the Recall@K@V values, thereby highlighting the nuanced interplay of these parameters in the evaluation process.

This innovative and advanced evaluation strategy facilitated the procurement of a more detailed and nuanced understanding of the model’s performance. The results of this experiment, which offer an extensive analysis of Recall@K@V evaluations, are depicted in Fig. 5. The strategic expansion of the traditional approach allowed for a thorough and informative dissection of the model’s performance.

Figure 5 illustrates the impact of modulating the voting threshold, termed “V”, on the task of determining a WSI’s primary diagnosis over a range of Recall@K values. The voting threshold specifies the number of top-retrieved items per patch taken into account in the decision-making process. By adjusting “V”, the breadth of information contributing to the final diagnostic decision can be fine-tuned, offering a nuanced perspective on the retrieval performance. The visual representation shows an initial trend of improved performance with an increasing voting threshold, underlining the value of a more inclusive decision-making process. Multiple “V” thresholds were examined to investigate this relationship, ranging from a conservative limit of 1 (i.e., only the top-retrieved item per patch contributes to the final decision) to a comprehensive limit of 22, encompassing all primary diagnoses in the database. The effects of these different thresholds are colour-coded for ease of comparison.

However, it is critical to note that there appears to be a limit to the benefit of this expansion. As demonstrated by the figure, a voting threshold greater than five signifies the beginning of an observable performance decline. This observed decline aligns with expectations, considering that the similarity scores across primary diagnoses tend to cluster after the top few candidates. Essentially, including too many lower-ranked diagnoses dilutes the influence of the most probable ones, leading to less accurate final decisions. Thus, a careful balance is required when choosing the voting threshold to optimally leverage the multi-voting scheme’s advantages while avoiding the pitfalls of over-inclusion.

Continuing to add votes past the optimal threshold tends to introduce excess noise into the voting system. This “over-voting” counter-productively decreases the precision of the voting mechanism and consequently reduces the final recall score. Hence, while expanding the voting pool can initially enhance diagnostic accuracy, careful moderation is necessary to prevent a decline in performance. The comparative performance of different methodologies on the GRH dataset, as measured by this particular metric, is detailed in Table 2. The “V” threshold within the Recall@K@V metric has been set to five for these evaluations. This setting was chosen as it consistently delivered the most advantageous results across different trials, as seen in Fig. 5. A clear observation from these comparisons is that the proposed LILE + H − DINO method excels beyond other approaches across varying recall thresholds. Notably, its superior performance holds up regardless of the recall threshold applied, further underscoring the robustness and generalization capability of the model. This reliable higher performance of the LILE + H − DINO model illustrates its potency and adaptability across diverse retrieval contexts in the GRH dataset.

### PatchGastricADC22 benchmark dataset

In the processing of the PatchGastricADC22 dataset which contains 991 WSIs from Mita hospital in Tokyo, Japan. All cases within this dataset have been diagnosed as adenocarcinoma and verified by three independent pathologists., patches of 300 by 300 pixels in size and 20X magnification were utilized. The description of each extracted patch was linked to the corresponding WSI it was obtained from. The primary distinction in this approach is manifested in the input selection for the model, which acts as an anchor for the training of the retrieval part. Instead of a center crop being traditionally employed, variability is introduced: for each iteration in the training process, a random crop of dimensions 224 by 224 pixels is chosen from the original 300 by 300 patch and inputted into the model. This methodology is based on the assumption that a subset of the original patch, specifically the 224 by 224 pixels crop, can adequately represent the accompanying descriptive text. Moreover, this method infuses a layer of randomness into the training process, potentially fostering an enhanced generalization ability for the model. However, during the test phase, a center crop is utilized. The overall model architecture was maintained in alignment with the configuration adopted for the GRH experiments. Pre-trained models from a classification task using the dataset featured in the “KimiaNet” paper^31^ were applied to both the student and teacher networks, while the BioBert^32^ was employed as the text encoder. The only variable in this arrangement was the number of trainable blocks in the vision component, where the last eight blocks were selected for adjustment. For the text encoder, the last two blocks were set as trainable.A learning rate of $5 \times 10^{-5}$ was applied, incorporating an adaptive adjustment strategy. This strategy involved reducing the learning rate by a factor of 0.5 upon detecting a plateau in the improvement of the evaluation metric (R@sum)., and the batch size was set at 64.

Random cropping with a size of 224 by 224 for extracted different views is applied. The data augmentation methodology implemented with the GRH dataset was mirrored here, with six Hyper augmentations ($\tau _H$) being applied to the student model and two less stringent ones ($\tau _M)$ to the teacher model. These augmentations include Random Crop, Random Vertical and Horizontal Flip, and Color Jitter (affecting Brightness, Contrast, Saturation, and Hue). The strength of these transformations was controlled, falling into either Hyper or Mild augmentation sets, depending on the desired extent of change. Optimal results were achieved when the parameters of the loss function were finely tuned, setting $\gamma$ to 1 and $\beta$ to 0.5. Given that the central goal of the model is to effectively locate and pair corresponding images and texts, this particular adjustment aids the model in concentrating on its main task.

Table 3 documents the performance of the model in patch-based and WSI-based retrieval compared to other methods. This table provides insights into the efficacy of the suggested method and its relative performance in comparison with other techniques.

**Table 3:** A comparison of the proposed method and other state-of-the-art approaches on the PatchGastricADC22 dataset, evaluating their performance in patch-based and WSI-based retrieval tasks.

Method	Text retrieval				Image retrieval				
	R@1	R@3	R@5	R@10	R@1	R@3	R@5	R@10	R@sum
Patch based									
BioMedCLIP (zero-shot)^35^	6.0	17.8	28.7	53.2	10.1	22.4	29.8	51.2	219.2
LILE^34^	18.2	37.8	49.1	78.2	28.5	46.9	48.3	68.2	375.2
LILE + DINO	19.6	40.4	53.8	80.0	34.2	50.6	55.0	70.0	403.5
LILE + H − DINO	**20.8**	**42.2**	** 54.8**	**81.3**	** 40.0**	**55.0**	**55.0**	**75.0**	**424.1**
WSI based									
BioMedCLIP (zero-shot)^35^	14.6	26.2	32.3	64.5	N/A	N/A	N/A	N/A	N/A
LILE^34^	32.4	46.1	64.3	85.8	N/A	N/A	N/A	N/A	N/A
LILE + DINO	35.0	49.5	66.2	88.3	N/A	N/A	N/A	N/A	N/A
LILE + H − DINO	**36.7**	** 52.3**	**67.8**	**93.2**	N/A	N/A	N/A	N/A	N/A

Best results highlighted in bold.

To evaluate the performance of the proposed approach and compare it with other methods, LILE is been used as the baseline and compared LILE + H − DINO with LILE + DINO and BioMedCLIP^35^ which is one of the *foundation models* for vision and vision language tasks that trained on large paired medical data. Notably, there are no pre-existing reported results for this dataset, which underscores the novelty of this research. Furthermore, for the sake of comparison, only the BioMedCLIP model was utilized in this instance due to its public availability and its standing as one of the SOTA techniques for cross-modal retrieval tasks in the medical field.

The same voting scheme proposed for the GRH dataset is applied for this dataset with a voting threshold of five for WSI-based retrieval tasks. The results show the efficacy of the combination of H-DINO in both patch-based and WSI-based tasks which outperformed all the other approaches. In this experiment, the pre-trained BioMedCLIP model^35^, which was trained on a large volume of figure-caption pairs extracted from biomedical research articles in PubMed Central, was employed without further fine-tuning. To facilitate the application of the BioMedCLIP model for WSI-based retrieval, the identical procedure used for other methods was diligently implemented. The results of BioMedCLIP, as expected, are the lowest among other approaches as it is only trained with images and text extracted from research articles, and it can show the importance of data in training models. The comparative evaluation of the proposed method, LILE + H − DINO, with LILE and LILE + DINO, offers some notable insights.

Among the methods charted in the table, a distinct performance advantage was exhibited by the proposed LILE + H − DINO. It outpaced both LILE and LILE + DINO at various recall thresholds, including R@sum, registering a lead of 48.9 and 20.6, respectively. This performance margin underscores the significance of incorporating self-supervised learning (SSL) and a tailored patching scheme in the proposed methodology, elements that played a pivotal role in enhancing retrieval accuracy.

On another note, a characteristic of this dataset is that each description is associated with all patches extracted from the corresponding WSI. Consequently, retrieving a WSI from a description is not feasible, as the correlation doesn’t lend itself to direct WSI retrieval. Therefore, the corresponding entries in Table 3 are marked as “N/A” (Not Applicable), indicating the inapplicability of certain metrics in this context.

The dataset under consideration contains authentic captions penned by pathologists, offering a practical application scenario for the proposed approach. Furthermore, the challenge escalates when distinguishing between descriptions and images pertaining to the same type of adenocarcinoma. The similarity in the nature of the images and text can blur the differentiation, thereby posing a considerable challenge. This is especially prominent when comparing the performance to that of the GRH dataset. The complex task of accurately matching the nuanced visual patterns in the images with the precise medical terminology in the captions can lead to reduced performance, reinforcing the intrinsic difficulty of the task. Nonetheless, successfully tackling this challenge can provide valuable insights for advancing cross-modal retrieval techniques in real-world clinical settings.

### LC25000 benchmark dataset

The LC25000 dataset was employed as another benchmark to evaluate the effectiveness of the proposed methodology. This dataset contains 25000 patches with the dimension of 768 by 768. All patches are in 20*X* magnification. In line with model requirements, these patches were resized to 448 by 448 pixels. The methodology previously detailed for the GRH dataset was adhered to, ensuring consistent operations across all datasets. As mentioned for the PatchGastricADC22 dataset, instead of the center 224 by 224 pixels crop feed into the model, a random crop has been fed to the cross-modal retrieval network as the same characteristic applied for the LC25000 dataset. One limitation that arose with the LC25000 dataset was the absence of information regarding the WSI from which the patches were extracted. This resulted in the reporting of *only patch-based retrieval outcomes* for this dataset.

The preparation of views for both the student and teacher networks was undertaken in an identical manner to previous operations for the GRH and PatchGastricADC22 datasets, including the number of views and augmentations that have been applied. An exhaustive and systematic experimental analysis has ascertained optimal parameter settings tailored for this dataset. In the context of the image encoder, the student and teacher networks, fine-tuned on the last six blocks of a pre-trained ViT trained on the dataset featured in the “KimiaNet” paper^31^, showed the best results. On the other hand, the last two blocks of BioBert^32^ were found to be most effective for the text encoder.

In terms of optimizing the loss function, the parameters were carefully calibrated. The parameter $\gamma$ was set to 1, while the parameter $\beta$ was set to 0.4, granting a slightly lesser emphasis to the SSL part in the loss function. A learning rate set at $5 \times 10^{-5}$ was adopted, with an adaptive reduction approach. This approach halved the learning rate when no further enhancements in the evaluation metric (R@sum) were observed., offering a good balance between training speed and model stability. The batch size was also set to be 64.

Comparisons were made between the performance of the proposed method and other existing methods. The insights derived from these comparisons have been collated and presented in Table 4. The findings affirm the proposed method’s robustness and effectiveness when applied to this benchmark dataset, supporting its validity across various scenarios. Notably, the MD^38^, CNN and SVM^39^, and MRFO and EO^40^ methods are unable to be applied in the image retrieval direction, and they do not provide results for R@3 and R@5. The absence of reported results for these models further highlights this limitation. The outcomes of this experiment are noteworthy and closely matched, largely due to the straightforward nature of the task, which involves recognition among merely five distinct categories. In this context, the distinguishing factor among various methodologies primarily lies in the R@1 metric (the strict classification case), given that all methods achieved a perfect score of 100 for R@3 and R@5. Consequently, R@10 has not been reported, considering that with only five primary diagnoses, any recall threshold above 5 would invariably yield a score of 100.

Despite the high level of competition, the proposed model, LILE + H − DINO, manages to inch ahead, albeit by a narrow margin. It surpasses the performance of MRFO and EO, as cited in^40^, by 0.2 for R@1, and outperforms LILE and LILE + DINO by 0.5 and 0.1, respectively, for R@1, which subsequently impacts the R@sum score.

This marginal yet significant superiority is particularly noteworthy given the circumstance where scores are concentrated near the ideal score of 100. The model’s ability to offer this subtle performance boost in a context where the scope for enhancement is minimal indicates its capacity to yield exceptional results in more complex scenarios.

Furthermore, image retrieval results have only been provided for architectures. This is because other approaches have only been capable of functioning in a single direction - specifically, classifying images based on their primary diagnosis. This further highlights the versatility and capability of networks in the context of image retrieval tasks.

**Table 4:** A comparison of the proposed method and other state-of-the-art approaches on the LC25000 dataset, evaluating their performance in patch-based retrieval tasks.

Method	Text retrieval			Image retrieval			
	R@1	R@3	R@5	R@1	R@3	R@5	R@sum
Patch based							
MD^38^	98.4	N/A	N/A	N/A	N/A	N/A	N/A
CNN and SVM^39^	94.0	N/A	N/A	N/A	N/A	N/A	N/A
MRFO and EO^40^	99.6	N/A	N/A	N/A	N/A	N/A	N/A
LILE^34^	99.3	100	100	100	100	100	599.3
LILE + DINO	99.7	100	100	100	100	100	599.7
LILE + H − DINO	**99.8**	100	100	**100**	**100**	**100**	**599.8**

Best results highlighted in bold.

## Discussion

Efficient, versatile retrieval across diverse modalities, like images and texts, is a key goal in cross-modal retrieval tasks for real-world applications. Scarce labelled data makes these tasks more challenging. To address this, a method to extract robust embeddings for both image and text modalities was proposed. The unique aspect of this approach is the integration of SSL within knowledge distillation for cross-modal retrieval tasks and an end-to-end training regime.

Central to the proposed framework is the emphasis on deriving rich feature embeddings from images through SSL, with a specific focus on the DINO architecture. We introduce a novel patching strategy, termed “harmonizing DINO” or H-DINO, tailored for pathology WSIs. This advanced method systematically uncovers and assimilates critical features across modalities, leading to the development of more robust embeddings. Such enriched feature representations are pivotal in enhancing performance across cross-modality tasks, demonstrating the significant impact of high-quality embeddings on retrieval effectiveness. Consequently, a refined methodology is developed that progressively identifies and captures the most important features across modalities, both in isolation and relative to one another.

A multi-head self-attention module enhances the network’s understanding of its modality, and the output is directed to a cross-attention module for aligning text and image modalities. An iterative alignment scheme using a memory network is employed in the proposed LILE + H − DINO strategy to augment features based on significant data segments highlighted by the self-attention and cross-attention modules. Moreover, A novel loss function is introduced, incorporating SSL and cross-modal retrieval objectives with weighted significance to balance these interconnected objectives.

The effectiveness of SSL in cross-modal retrieval is demonstrated, and the integration of tailored patching for WSIs enhances performance. Harmonizing patching preserves contextual integrity and structural consistency, contributing to richer image interpretation. Performance differences between LILE, LILE + DINO, and LILE + H − DINO illustrate the benefits of merging SSL and the tailored patching scheme.

The proposed methodology gains significance from its application across varied datasets, including the challenging GRH breast cancer dataset, which features 22 primary diagnoses and presents significant hurdles due to its diagnostic range and subtle anatomical differences. Its real-world value is highlighted through its use on a dataset of pathologist-composed images and descriptions, showcasing its practical utility. Additionally, the method’s versatility and effectiveness are affirmed by its successful performance on a simpler dataset with just five primary diagnoses, underscoring its adaptability and wide-ranging applicability.

However, a potential avenue for future research lies in evaluating the retrieved images and texts through the lens of content similarity. Solely interpreting the results based on the categories of retrieved content is insufficient. This approach underscores the need for a more nuanced analysis that accounts for the intricate relationships and similarities between the contents, thereby providing a comprehensive understanding of the model’s performance. Another promising direction for future research involves incorporating diverse clinical information or additional data modalities into cross-modality tasks, enhancing the comprehensiveness and applicability of the analysis.

## Method

The proposed methodology is integrated with several critical components, collaboratively aimed at addressing cross-modality retrieval challenges in the pathology domain. Detailed insights into their design and operation will be provided in subsequent sections.

**Figure 6:**
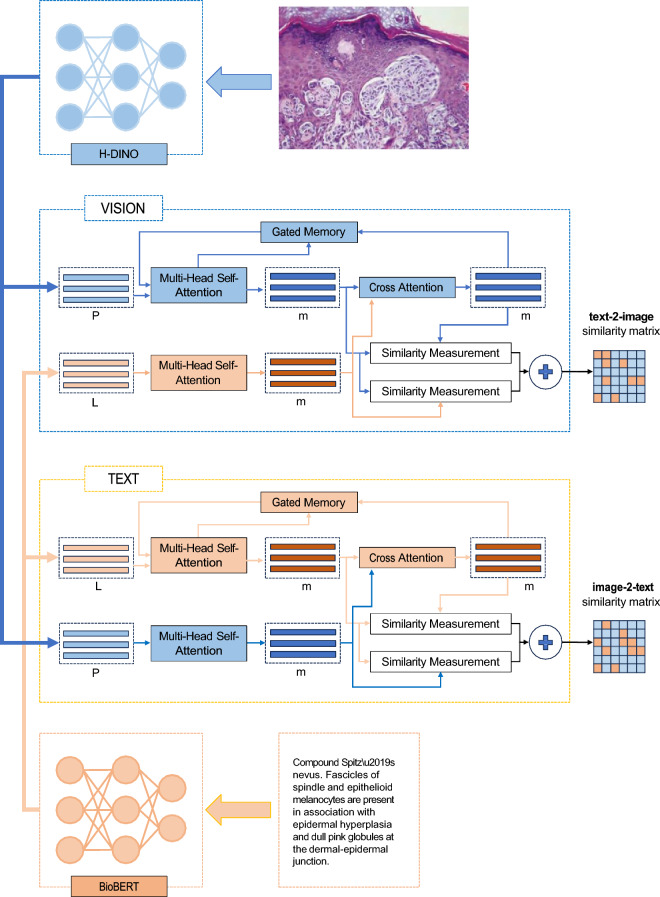
The architecture of H − DINO + LILE for image-text data is shown. It comprises H-DINO architecture and BioBERT to extract features for both vision and text modalities and LILE backbone to align feature representation of pair images and text.

The architecture from LILE^34^ which designed for cross-modality tasks is utilized as the backbone of the proposed network. The Transformer architecture is employed for both image and text encoding backbones, with self-attention modules being leveraged to highlight key features of images and text. Additionally, in line with LILE^34^ and other methodologies^6,7^, cross-attention modules are employed to extract important segments from images and text, considering their relevance to the other modality, namely text and images, respectively. Moreover, as previous studies^6,34^ suggested, a gated memory is applied to refine extracted features for images and text regarding the output of the cross-attention module in an iterative scheme. The architecture of the proposed method is depicted in Fig. 6.

As was highlighted in the introduction, the scarcity of adequately labelled data for model training is recognized as a prevailing challenge in the medical field^41^. This challenge is notably apparent in the visual modality, even though numerous dependable medical language models recently have been introduced^32,42^. To address this, an self supervised learning (SSL) paradigm to derive image features is introduced in this study. Within the proposed network, the DINO approach^27^ has been adapted by introducing a unique patching technique named harmonizing DINO, abbreviated as H-DINO, for WSIs. However, other SSL approaches such as CTransPath^43^ can be modified and applied in here as well. This technique is further detailed in the patching section.

The DINO framework authors suggested, creating various crops from a single image^44^. This resulted in set *V*, holding multiple views of the image, including two global and several local views at reduced resolution.

**Figure 7:**
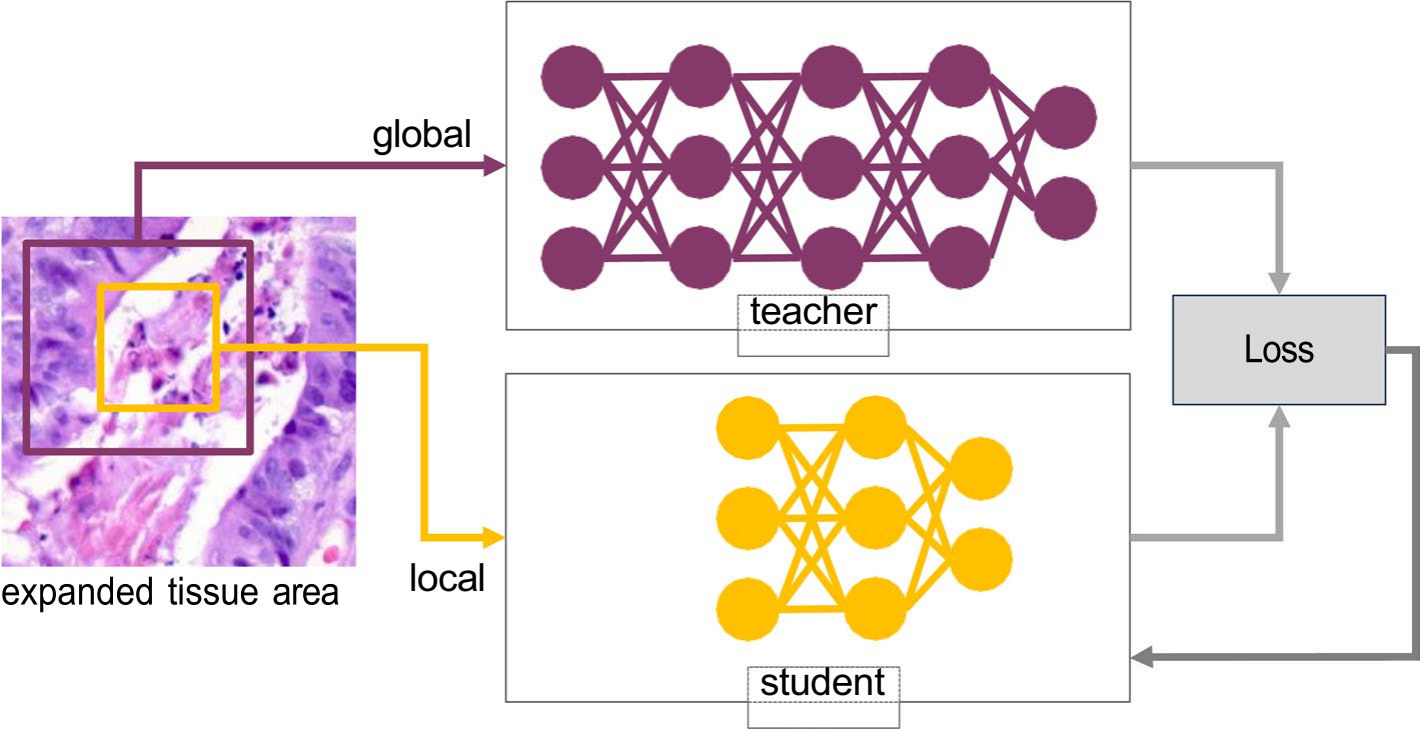
Diagram of the H-DINO Architecture: Illustrating both the student and teacher networks. For the sake of clarity, only one patch per view is represented.

Considering WSI’s unique needs and patch extraction in histopathology, the set *V* was revised. The original DINO paper used 96 × 96 local patches and 224 × 224 global ones. Yet, altering the magnification of patches from a WSI at 20x, common in digital pathology, could introduce inconsistencies. To mitigate this, a tailored patching solution named H-DINO, aligning with WSI techniques, is introduced. The architecture is shown in Fig.7, with further details in the patching section.

### Patching

**Figure 8:**
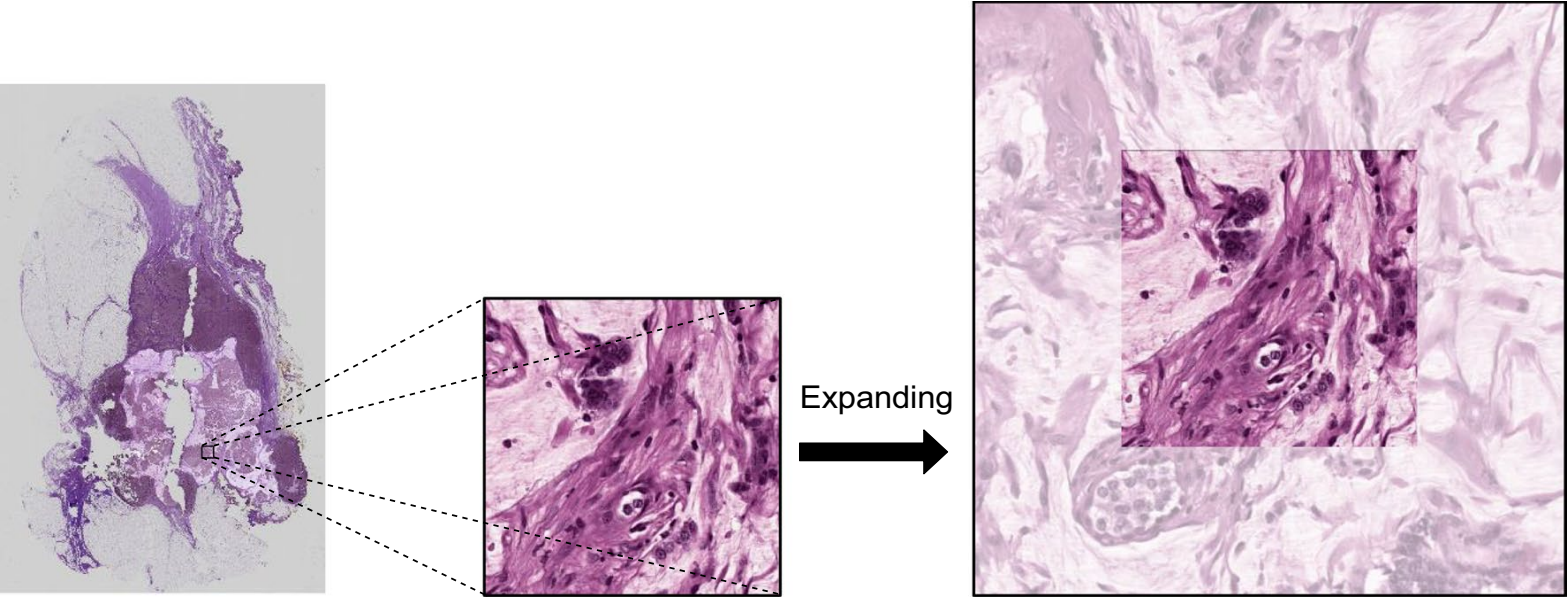
Patch extraction and expansion from a WSI: The process begins with mask overlay, patch extraction, expansion, and transformation.

In pursuit of this extension to DINO, an approach has been adopted where the extraction of patches is carried out on a scale larger than the final size intended to be inputted into the network. This is accomplished by enlarging the patch size around the specific location based on the dataset and patching method. The visualization of this step can be seen in Fig. 8. Subsequently, two categories of views—more global and more local—are extracted from the expanded patch, with each being subjected to its respective set of transformations. The distinction between these views is fundamentally determined by the size of the cropped region. The cropping range for the more local views is set between 50 and 140, while for the more global views, the cropping size is set between 140 and 224. Given that uniformity in magnification is maintained in all selected patches, the core features of these patches remain unaltered. This departure from the DINO’s patching approach is crucial for computational pathology. This consistency in the patches magnification ensures that each patch holds its unique representation and characteristics intact, which in turn assists in maintaining the consistency of the information it offers.

Moreover, the uniformity in magnification also guides the network’s learning process in a meaningful way. By processing patches that are expected to share similar attributes, the network learns to map related concepts or visual features closer to each other in its internal representation space. This capacity is expected to be central to the network’s ability to distinguish between different classes and categories and, thereby, crucial to achieving high performance on classification or retrieval tasks in the forthcoming experiments.

**Figure 9:**
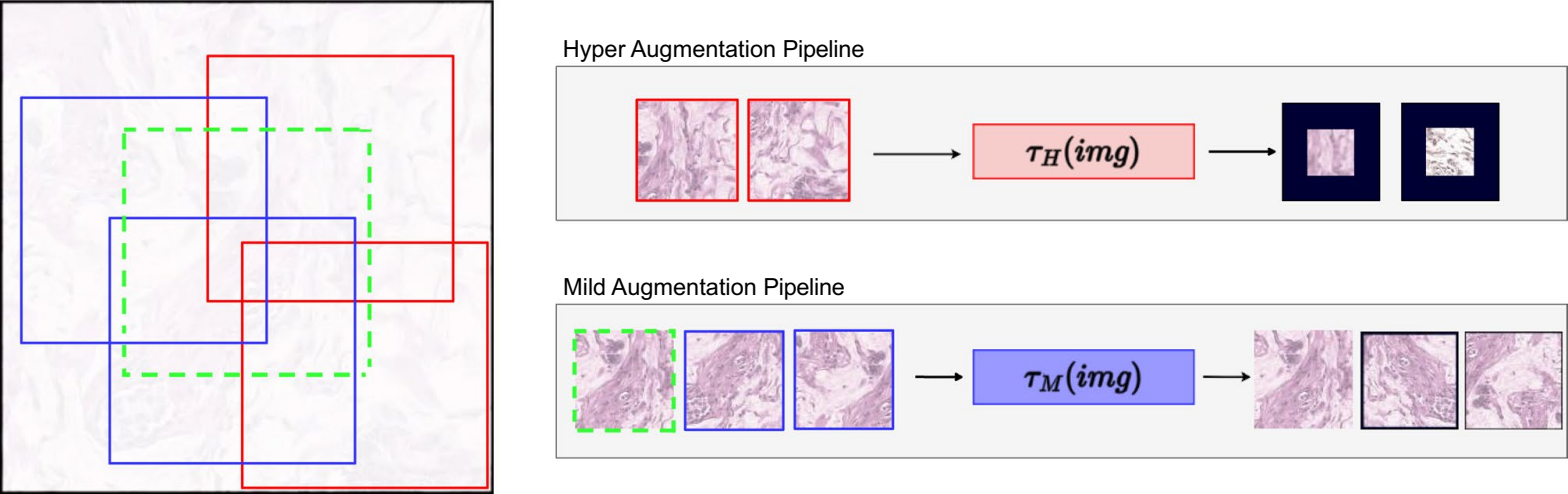
View Selection and augmentation pipeline: left shows random view selection for Hyper ($\tau _{H}$) and Mild ($\tau _{M}$) augmentation, right displays their respective pipelines with varied intensity levels.

These transformations are carefully selected to match the specific network into which the patch will be integrated. A comprehensive visualization of this entire process can be found in Fig. 9. This figure depicts the interplay among the extraction, enlargement, and transformation of patches, all contributing to the distinct approach that has been adopted in the design.

After the patch selection, the more robust and less noisy patches are passed through the teacher model, while all the patches that have been generated in the patch selection stage are passed through the student model. In the proposed framework, as delineated in the DINO paper^27^, both the teacher and student networks share an identical architectural design, yet they are characterized by distinct sets of parameters: $\theta _s$ for the student network and $\theta _t$ for the teacher network. The training process for these networks is conducted concurrently, adhering to the methodology outlined in Eqs. (6) and (7).

In addition to deriving robust feature representations for images, feature representation for the text modality must also be extracted. Consequently, a Transformer architecture was employed, as suggested by the authors of the BioBERT paper^32^. The selection of BioBert as the text encoder is justified by its comprehensive training on a broad collection of medical literature, including content related to pathology. However, a potentially superior alternative could be the adoption of a model that has been explicitly trained on a substantial compilation of pathology reports.

### Loss function

The proposed loss function guides the training of the entire model in an end-to-end paradigm which helps the network to have better performance and more flexibility^45^. It means that the proposed loss function should contain two parts, one set of loss functions specifically designed for training the cross-modal retrieval model.and another loss function dedicated to training the SSL network and another

The first part should draw paired image-text data closer within the shared space while making the unpaired data far from each other.

For each iteration step $k$, the similarity score between an image *I* and text *T* is determined in a manner akin to the method proposed by LILE^34^:

1 $$\begin{aligned} \begin{aligned} S_k(I,T) &= \alpha \left( \frac{1}{m}\sum _{i=1}^{m} S_k^{{(v,v\xrightarrow []T})}(v_i,T)+\frac{1}{m}\sum _{i=1}^{m} S_k^{{(w,w\xrightarrow []I})}(I,w_i)\right) \\ &+(1-\alpha )\left( \frac{1}{m}\sum _{i=1}^{m} S_k^{(v,T)}(v_i,T) + \frac{1}{m}\sum _{i=1}^{m} S_k^{(w,I)}(I,w_i)\right) \end{aligned} \end{aligned}$$

In the equation above, $\alpha$ is a scalar weight parameter that moderates the influence of the similarity score components. $S^{{(v,v\xrightarrow []T})}(v_i,T)$ and $S^{{(w,w\xrightarrow []I})}(I,w_i)$ are defined as the similarity score between image regions and text *T*, and text tokens and image *I*, respectively.In the evaluation of similarity between image and text embeddings, a diverse array of metrics can be utilized. However, drawing from insights in recent studies^35,35,46,47^, cosine similarity emerges as the preferred method due to its effectiveness and alignment with recent findings in the field and is applied to the suggested solution. These similarity scores are calculated as

2 $$\begin{aligned} S_k^{{(v,v\xrightarrow []T})}(v_i,T)&= {{\mathbf {cosine \,similarity}}}(v_i, A_k^v),\\ S_k^{{(w,w\xrightarrow []I})}(I,w_i)&= {{\mathbf {cosine \,similarity}}}(A_k^t, w_i). \end{aligned}$$

Where each element of $A^x$ captures the most important parts of the corresponding $x_i$ from modality *X* which can be image or text given the other modality as context.

Further enhancements in the similarity score can be achieved by incorporating $S^{(v,T)}(v_i,T)$ and $S^{(w,I)}(I,w_i)$. These two terms are expressed in

3 $$\begin{aligned} S_k^{(v,T)}(v_i,T)&={{\mathbf {cosine \,similarity}}}(v_i, T),\\ S_k^{(w,I)}(I,w_i)&= {{\mathbf {cosine \,similarity}}}(I, w_i). \end{aligned}$$

This inclusion allows the model to maintain the semantic meaning of each instance while concurrently striving to minimize the distance between paired instances. To consolidate all *k* steps, the final similarity score between image I and text T is derived as

4 $$\begin{aligned} S(I,T) = \sum _{k=1}^{K} S_k(I,T). \end{aligned}$$

Here *k* is the number of matching steps that will be set as a hyper-parameter.

Ultimately, the cross-modality retrieval loss is computed as

5 $$\begin{aligned} {\mathcal {L}} = \left[ \Delta - S(I_i, T_i)+S(I_i, T_j)\right] _+ + [\Delta - S(I_i, T_i)+S(I_j, T_i)]_+. \end{aligned}$$

This strategy has been widely recognized and applied in image-text matching domains, with numerous studies demonstrating its effectiveness^11,48–50^. In Eq. (5), $\Delta$ is a margin, and $[x]_+ = max(0,x)$. *S*(*I*, *T*) from Eq. (4) gauges the similarity between image *I* and text *T*, forming a symmetric similarity matrix *S* of size $n\times n$, where *n* is the mini-batch size during training.

Meanwhile, the second part of the loss function aims to force the vision model to generate a more robust feature representation for the given images using an SSL method as described in DINO paper^27^. In the training procedure, the loss will be minimized with respect to $\theta _s$:

6 $$\begin{aligned} \min _{\theta _s} \sum _{x \in \left\{ x_1^M, x_2^M \right\} }^{} \sum _{\begin{array}{c} x'\in V \\ x' \ne x \end{array}}{-P_t(x)log(P_s(x^{\prime }))}. \end{aligned}$$

The proposed patching scheme generates multiple views of a given image, which are assembled into a set *V*. This set is characterized by the inclusion of two distinct subsets of image views, differentiated by the type of augmentation applied. One subset comprises views generated through the application of “Mild Augmentation” ($\tau _M$), while the other uses “Hyper augmentation” ($\tau _H$).

In the context of Eq. (6), *x* is a selection made from the subset of views created through the $\tau _M$ technique. In contrast, $x^{\prime }$ is chosen from the collective set of all views, excluding the specific view selected for *x*. Eq. (6) seeks to minimize the distribution distance between these various views of the same image, which is a critical step towards achieving a more robust image understanding. This minimization process ultimately assists the model in distinguishing subtle differences within the image and understanding how different elements of the image relate to each other.

The proposed loss function here can be applied on any number of patches; however, as the authors in the DINO paper have suggested, the proposed method only used two patches for the teacher network. In the approach under discussion, the absence of an initial teacher model, denoted by $g_{\theta _t}$, necessitates its iterative construction from previous versions of the student network. As suggested by the authors in the DINO paper, an approach that remarkably aligns with this framework involves applying an EMA to the student weights and a strategy often referred to as a momentum encoder. The update rule for this strategy, as described in the DINO paper^27^, is given by

7 $$\begin{aligned} \theta _t \leftarrow \lambda \theta _t + (1-\lambda )\theta _s, \end{aligned}$$

where $\lambda$ undergoes a gradual transition from 0.996 to 1 during the training process, following a cosine schedule. This dynamic tuning of $\lambda$ orchestrates the continuous updating of the teacher network parameters ($\theta _t$) based on the concurrently evolving student network parameters ($\theta _s$). This symbiotic process effectively transfers learned representations and knowledge between the teacher and student networks, promoting consistent learning progress and model robustness.

After calculating the loss functions for the self-supervised task based on Eq. (6) and the cross-modal task based on Eq. (5), the total loss is computed as follows:

8 $$\begin{aligned} {\mathcal {L}}_{total}&= \beta {\mathcal {L}}_{ssl} + \gamma {\mathcal {L}}_{cross-modal}, \end{aligned}$$

9 $$\begin{aligned} {\mathcal {L}}_{ssl}&= \sum _{x \in \left\{ x_1^M, x_2^M \right\} }^{} \sum _{\begin{array}{c} x'\in V \\ x' \ne x \end{array}}{-P_t(x)log(P_s(x^{\prime }))}, \end{aligned}$$

10 $$\begin{aligned} {\mathcal {L}}_{cross-modal}&= [\Delta - S(I_i, T_i)+S(I_i, T_j)]_+ + [\Delta - S(I_i, T_i)+S(I_j, T_i)]_+. \end{aligned}$$

The weight parameters, denoted by scalars $\beta$ and $\gamma$, play a crucial role in determining the impact of each loss term. They regulate and moderate the influence of these terms on the overall loss function ${\mathcal {L}}_{ssl}$.

## Data Availability

Some datasets analysed during the current study are available in the PatchGastricADC22 repository, https://zenodo.org/records/6021442, and in LC25000 repository, https://github.com/tampapath/lung_colon_image_set. The GRH dataset from Grand River Hospital (Kitchener, ON, Canada) analysed during the current study is not publicly available due patient privacy guidelines at GRH.
